# Configuration of prosocial motivations to enhance employees’ innovation behaviors: From the perspective of coupling of basic and applied research

**DOI:** 10.3389/fpsyg.2022.958949

**Published:** 2022-09-28

**Authors:** Yuting Lu, Linlin Zheng, Binghua Zhang, Wenzhuo Li

**Affiliations:** ^1^Fujian Academy of Social Sciences, Fuzhou, China; ^2^Business School, Huaqiao University, Quanzhou, China; ^3^School of Architecture and Urban-Rural Planning, Fuzhou University, Fuzhou, China; ^4^Business School, Hohai University, Nanjing, China

**Keywords:** prosocial motivation, research behavior, transformation behavior, qualitative comparative analysis, configuration

## Abstract

Prosocial motivation refers to the employees’ willingness to invest for the sake of helping others. It improves basic and applied research behaviors of employees and the interaction between them. Employees’ innovation behavior depends on prosocial motivation because the motivation to protect the interests of others may promote knowledge sharing and knowledge coupling. However, there is a research gap in solving the optimal solution of prosocial motivations that facilitates different types of innovation behaviors based on the combination of prosocial motivations. We perform a qualitative comparative study on the effect of the motivation configurations on innovation behaviors. We find that highly basic and highly applied research behaviors share in common collectivism-based, principlism-based, contextual, and situational motivations which work in all configurations. But the core conditions between the two are different, which are principlism-based and situational motivations, respectively. In addition, both highly basic-to-applied and highly applied-to-basic transformation behaviors share the same core condition and the same secondary conditions with highly basic and highly applied research behaviors, respectively. Moreover, the behaviors of non-highly basic research and non-highly basic-to-applied transformation share the severe absence of egoism-based motivation as the core condition in common. Non-highly behaviors of applied research and applied-to-basic transformation have a common point of the severe absence of the pressure-based type as the key. Finally, we also analyze active and passive prosocial degrees of all types of high/non-high innovation behaviors. Our study deepens the academics’ thinking on multi-dimensional prosocial motivation and the classification management of coupling innovation behavior and provides implications for practice.

## Introduction

With the development of science and technology, basic and applied research departments produce dual characteristics of independence and openness gradually. It puts forward high requirements on the behavior management of basic and applied research departments. In particular, the coupling process of the two also puts forward high challenges to innovation behavior management. However, the correlation between the two remains low. The innovation behaviors can no longer meet the requirements of a highly differentiated and integrated knowledge production mode (Liu et al., 2022). Therefore, the coupling of basic and applied research has become an important force to cope with changes (López-Martínez et al., 1994; Nagane and Sumikura, 2020). Employees who take part in the innovation activities are the main force of original innovation. How to use the complex psychological characteristics, especially like prosocial motivations of employees in innovation departments to manage the coupling behaviors, is an important topic.

Prosocial motivation refers to employees’ willingness to invest for the sake of helping others (Batson, 1987). It is considered one of the key factors affecting employees’ or organizational creativity and employees’ innovation ability (Gebauer et al., 2008; Hoever et al., 2012; Li and Bai, 2015; Pian et al., 2019). With the increasing complexity and uncertainty of innovation, the research presents two challenges. First, prosocial characteristics of single motivation have limitations in explaining employees’ innovation behavior (Li and Bai, 2015; Shie et al., 2022). Employees may not only hope that they can be free from life pressure and working environment to show their innovation ability but also hope that their innovation behaviors can be improved by their abilities, characteristics, and external situation (Sun Y. et al., 2020). Prosocial characteristics with different motivations can reflect employees’ structural characteristics. Second, there is an insufficient discussion on the impact of prosocial motivation on the composition of innovation behaviors (Bertels, 2018). Therefore, it is necessary to further explore the applicability and effect of prosocial motivation on the innovation behavior within the department and the innovation behaviors between the departments. So, the prosocial way to maximize employees’ innovation potential is to improve basic and applied research behaviors and their coupling merits attention. Configuration of prosocial motivations provides a new perspective. The optimal solution of prosocial motivations that facilitates innovation potential based on the combination of different personalities and states should be explored.

## Literature review and theoretical basis

### Literature review

Employees with prosocial motivation are altruistic (Batson, 1987). They tend to think about what is useful to colleagues, superiors, and organizations and are willing to help others actively (De Dreu and Nauta, 2009; Grant and Berry, 2011). It will help them generate new ideas beneficial to others and achieve the innovation behaviors (Grant, 2007; Grant and Mayer, 2009; Grant and Berg, 2011; Hughes et al., 2018). It can also enhance the influence of intrinsic motivation on creativity (Kunda, 1990; Caruso et al., 2006; Anderson et al., 2014; Thuan and Thanh, 2020). Specifically, it is reported that prosocial motivation can help employees eliminate limitations from their aspect and focus on others and organizational levels (Hoever et al., 2012). It can help employees generate useful ideas with a high degree of novelty and promote their communications with leaders, which can bring benefits to others or organizations and finally improve innovation performance (Bear and Hwang, 2015; Kim and Choi, 2018; Che et al., 2019). Then, it is pointed out further that when prosocial motivation increases, employees are more concerned with collective interests and more willing to share and to participate in team innovation. Prosocial characteristics stimulate employees’ sharing knowledge with others or organizations, which greatly enhances personal job autonomy and organizations’ innovation ability (Pian et al., 2019; Tian et al., 2021; Lin et al., 2022). Collective prosocial motivation reduces knowledge hiding in teams and is conducive to promoting innovation behaviors (Babic et al., 2018). But when employees perceive greater external pressure, it is not conducive for them to sharing knowledge (Škerlavaj et al., 2018).

However, the real prosocial psychology of employees cannot be reflected by single prosocial motivation, otherwise, it will affect the validity of its explanation (Li and Bai, 2015; Shie et al., 2022). So, academics began to pay attention to the influence of multi-dimensional prosocial motivations on employees’ innovation behavior. Li and Bai (2015) considered the difference between employees’ prosocial motivation and intrinsic motivation and found that prosocial motivation could amplify the positive impact of employees’ internal motivation on their creativity. But when the policies endow the employees with a stable environment and make them feel abundant, employees are less likely to be driven by prosocial motivation, but by internal motivation (Jeong and Alhanaee, 2020). Zee et al. (2020) paid attention to explicit and implicit characteristics of prosocial motivations and found that employees with explicit prosocial motivations show more creativity. Gohler (2021) distinguished the prosocial motivations of principlism and collectivism and found that both have positive effects on knowledge sharing significantly. In addition, considering the influence of knowledge diversity on innovation behavior, Sun Y. et al. (2020) reported that prosocial interaction helps innovators overcome the problem of knowledge diversity. Besides, Bertels (2018) explored the difference between the influence of prosocial motivation on novelty and the influence of prosocial motivation on the usefulness of creativity. He reported that members who received an assignment description that included opportunity framing produced more novel solutions, whereas those who received the same assignment but with prosocial framing created less useful solutions. The difference between the effect of active knowledge exchange on innovation behavior and that of passive knowledge exchange on it was also focused on by Mittal et al. (2020). They brought the prosocial, proactive exchanges to the forefront of knowledge exchanges, which predominantly focused on reactive knowledge exchanges.

Therefore, it is necessary to decompose the type of innovation behaviors and to explore the influence of prosocial motivations on them from the perspective of multi-dimensional prosocial characteristics. On one hand, the influence of prosocial motivations is a complex process and innovation behavior is more susceptible to the prosocial characteristics of multiple motivations (Gohler, 2021). How to solve the optimal solution of prosocial motivations that facilitate different types of innovation behaviors needs to be further explored. On the other hand, previous analyses of the internal composition and characteristics of innovation behavior are insufficient (Bertels, 2018). According to the output process of innovation, innovation behaviors can be specifically divided into basic research behavior, applied research behavior, basic-to-applied transformation behavior, and applied-to-basic transformation behavior. How to overcome the difference between the prosocial motivations of employees who take part in innovation activities within the departments and those of the employees between the innovation departments also needs to be explored further. As such, this study explores the effect of multi-motivated prosocial combinations on multiple types of innovation behaviors.

### Theoretical basis

#### Index system of prosocial motivation

Prosocial motivation is related to employees’ personality traits and psychological states, and how they interact with each other. The prosocial characteristics of multi-motivation can better show the real states of employees’ psychological cognition. Personality traits and states are important summaries of the performance of prosocial motivation (Grant, 2008). To reflect the multi-dimensional prosocial characteristics of employees, Vallerand (1997) divided them into global, contextual, and situational types. Gebauer et al. (2008) classified them into pleasure-based and pressure-based motivations at the interior and exterior of driving forces. They were further divided into egoism-based, altruism-based, collectivism-based, and principlism-based motivations from the perspective of the purpose of motivation (Batson et al., 2011). These three ways to divide it can get eight types of prosocial motivations, which can reflect almost all aspects of prosocial motivations for personality traits and psychological states.

According to the motivated information processing theory, social motivation affects the content and the direction of information processing, and the desires of individuals can shape the way they react to information (De Dreu, 2006; Nijstad and De Dreu, 2012). Generally speaking, intrinsic motivation gets the closest way to the individual’s desire, which is the lowest degree of information processing for the individual. When employees’ prosocial motivation is closer to intrinsic motivation, they are more likely to connect the experiences of others with their own and empathize with others, show concern for others, and identify with the experiences of others (Aron et al., 1991; Sun J. et al., 2020). In other words, the closer the employees’ desire is to the extrinsic motivation, the higher the employees’ degree of information processing, and the lower the employees’ efficiency in making prosocial decisions. Based on it, we divide prosocial motivation into actively and passively prosocial motivations, considering the difference between initiative and passivity of prosocial motivations.

Active prosocial motivations are based on value orientation internalized, including pleasure-based, altruism-based, collectivism-based, and principlism-based types. Among them, pleasure-based motivation is the desire to benefit others, which is motivated by a sense of happiness and the healthy development of body and mind. It is mainly expressed as an emotional state (Habashi et al., 2016). The collectivism-based prosocial motivation is the motivation to maximize collective interests from the perspective of the entirety. It is mainly motivated by one’s contact with the members in need of help so that employees can focus on how to protect the state of the collective interests (Luria et al., 2015). Both can be expressed as the psychological state of the individual. The altruism-based type is the desire to benefit other people or groups (Eisenberg et al., 2016). The principlism-based motivation refers to a stable personality trait with an outlook on prosocial life and values, which attaches great importance to the interests of others and groups. Both can be expressed as relatively persistent characteristics, which can be shown as a personal trait of altruism.

Passive prosocial motivations mainly focus on motivation constrained by target orientation and time focus, including pressure-based, egoism-based, contextual, and situational motivations. Among them, pressure-based motivation is the form of motivation to fulfill obligations (Škerlavaj et al., 2018). Contextual motivation focuses on employees’ motivation toward a specific domain or class of behavior and is moderately variable across time and situations (Rodrigues et al., 2017). Situational motivation focuses on employees’ motivation toward a particular behavior at a particular moment in time, which is more specific but unstable (Rodrigues et al., 2017). These three kinds of prosocial motivations can be regarded as extrinsic motivations, which are mostly expressed as a psychological state. It can help employees focus on protecting the interests of others due to external drive. Egoism-based motivation is the motivation to help others from one’s interests. It can be shown as a personality trait (Eisenberg et al., 2016). Specific indicators of prosocial motivations are constructed as shown in Table 1.

**TABLE 1:** Index system of prosocial motivation.

First-level index	Secondary index	Interpretation of index
Prosocial motivation for initiative	Pleasure-based motivation	Motivated by the sense of happiness and the healthy development of body and mind
	Altruism-based motivation	The motivation to help others as the ultimate goal
	Collectivism-based motivation	Motivation to maximize group interests
	Principlism-based motivation	Have a stable personality tendency of prosocial outlook on life and values
Prosocial motivation for passivity	Pressure-based motivation	Motivation to fulfill obligations
	Egoism-based motivation	Motivation to help others for your own gain
	Contextual motivation	Motivation toward a specific domain or class of behavior
	Situational motivation	Motivation toward a particular behavior in a particular moment in time

#### Characteristics and index system of innovation behavior

Innovation behavior refers to the process in which basic and applied research communities can acquire or create knowledge by internal cooperation or cross-cooperation and constantly generate new knowledge. In the process of internal cooperation, homogeneous innovation behavior comes into being. It refers to the behavior of members in a basic research department to make a breakthrough in the basic theory within the department or with members in other basic research departments. It also refers to the behavior of members in the applied research department to make a breakthrough in the technological application within the department or with members in other applied research departments. So, homogeneous innovation behavior includes basic and applied research behaviors. Specifically, basic research behavior refers to the behavior of a few basic researchers who have complementary theoretical knowledge and are willing to assume mutual responsibilities for common research purposes, which is driven by research projects with the main function of academic innovation (Xia and Yang, 2020). Applied research behavior is a type of research behavior with a clear direction and industrial technology breakthrough that can be achieved in a relatively short period. In the cross-cooperation, heterogeneous innovation behavior takes place. It refers to the behavior of integrating basic and applied knowledge to improve the structure of innovation and to use the advantages of differentiation in research and development of others by matching basic and applied research subjects. It contains basic-to-applied and applied-to-basic transformation behaviors. The former gets at the behavior that makes technological breakthroughs based on existing findings and theories. The latter gets at revealing the essence of objective things and the law of movement from applied research achievements.

The common members who are involved in the activity of basic research and basic-to-applied transformation include tutor graduate students and teachers-teacher research teams, whose members have a high level of knowledge with a knowledge structure that is relevant and complementary (Smith, 1971). Moreover, the age structure of the members is reasonable, which leads to the characteristics of the coexistence of experts’ experience and young people’s passion. There are a large number of postgraduate members who are the leading force in teams. They are in a period of highly active thinking, without obvious experience constraints. Therefore, they are more innovative, centripetal, and energetic. To some extent, it is similar to the characteristics of the members participating in applied research and applied-to-basic transformation activities. However, the differences between basic research behavior, applied research behavior, and transformation behaviors are mainly reflected in three aspects, namely the goal of innovation behavior, behavioral stability, and behavioral sustainability.

The differences in the target among innovation behaviors are mainly manifested in the following four sub-items. (1) Compared with members participating in applied and applied-to-basic research, basic and basic-to-applied research members do not consider the market prospects of achievements and their market awareness is indifferent (Nejati and Shafaei, 2018). The basic and basic-to-applied research activities they engage in are relatively independent. They can choose research topics relatively freely, formulate research plans based on their interest and capabilities, and achieve knowledge innovation through basic theoretical research. (2) Members who take part in basic and basic-to-applied research focus on the pursuit of spiritual needs. Members with high quality and common goals have a strong sense of honor and accomplishment. Their spiritual needs, such as obtaining social respect and maximizing self-realization, exceed material needs to a certain extent (Zhao et al., 2014). But members who participate in applied and applied-to-basic research do not. (3) Members who take part in basic and basic-to-applied research emphasize academic equality. Every member focuses on mutual respect and trust and creating a democratic team atmosphere to give full play to organization cohesion and to improve the overall innovation strength. However, members in applied and applied-to-basic research can accurately refine and decompose research goals and tasks and give everyone corresponding powers and responsibilities based on the effective division of labor to achieve research goals. (4) Basic and basic-to-applied research members can coordinate behavior and conduct equal academic transactions. This wins transaction partners with its own “transaction” value and obtains other supplementary capabilities (Zhao et al., 2014; Bordogna, 2020). The essence of this process is that the behavior of members is coordinated, rather than subordinate and mutually exclusive. Applied and applied-to-basic research behaviors rely on members to provide value to other members in collaboration to realize the actual value of their behavior.

In addition, members in applied and applied-to-basic research have relatively concentrated goals, and the organizational structure is hierarchical. Conversely, the organizational structure in basic and basic-to-applied research departments is like a relatively stable network structure in which members and tasks are interconnected and dependent (Tierney and Farmer, 2002; Zou, 2019). Moreover, knowledge sharing requires weakening the hierarchy cognition of participants, enabling members to communicate on a more equal basis and forming a “peer-to-peer” knowledge network, which helps members interact with each other.

Finally, basic and basic-to-applied research behaviors that can produce significant research achievements form a research entity naturally based on long-term cooperation (Liang and Zhu, 2002; Popova et al., 2017). These types of research behaviors have the characteristics of relatively loose requirements for research task. Once basic and basic-to-applied research teams are established, they should be persistent and close and be able to do in-depth and continuous work around relevant research directions. Basic-to-applied and applied-to-basic transformation teams are usually temporary for a certain research task. Once the transformation achievements are obtained or their application enters a mature stage, the teams are dissolved. It is not conducive to knowing each other’s expertise, forming a working tacit understanding, and accumulating research knowledge and experience (Portes, 2010). The specific construction of innovation behavior is shown in Table 2.

**TABLE 2:** Index system of innovation behavior.

First-level index	Secondary index	Interpretation of index
Homogenous innovation behavior	Basic research behavior	Behaviors of basic researchers who are willing to assume mutual responsibilities for common purposes, which is driven by basic research projects and can realize academic innovation
	Applied research behavior	Behaviors with clear direction and industrial breakthrough that can be achieved in a relatively short period of time
Heterogeneous innovation behavior	Basic-to-applied transformation behavior	Research behavior that makes technological breakthroughs based on existing findings and theories
	Applied-to-basic transformation behavior	Theoretical research behavior revealing the essence of objective things and law of movement in applied research

#### Theoretical model

Based on the difference in the characteristics of different innovation behaviors, it is assumed that the effect of prosocial motivations on different types of innovation behaviors is also different. Due to the low cost of homogeneous knowledge innovation, members of basic and applied research departments tend to carry out innovation activities in a simple homogeneous environment. So, the active prosocial motivations generated from different degrees of internalized value orientation will play an important role. The tendency of altruistic behaviors promotes the sharing of scientific or technological knowledge within the departments, which is conducive to the realization of homogeneous innovation behaviors. Generally speaking, the demand for basic research achievements often changes with the demand for the key technologies, and it is always out-of-market demand. The characteristic may submit a high claim regarding the role of specific prosocial motivation. Compared with the single prosocial motivation, prosocial motivations with multi-dimensional initiative can more effectively explain the occurrence of homogeneous innovation behaviors.

Furthermore, due to the cross-sectoral difficulty, the execution of heterogeneous innovation activities not only needs effective communication between departments but also bears the cost effect of heterogeneous knowledge fusion. Therefore, high technical requirements are put forward for prosocial motivations for initiative and passivity. Compared with homogeneous innovation behaviors, the realization of heterogeneous innovation behaviors requires not only the presentation of an active prosocial state and the explicit prosocial initiative but also the constraint effect of passive prosocial motivation to conditionally realize the knowledge transformation behaviors.

**FIGURE 1:**
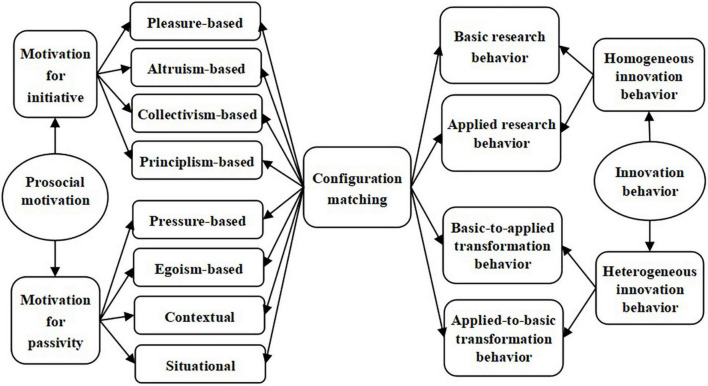
Theoretical model of the effect of multidimensional prosocial motivations on different types of innovation behaviors.

Hence, it can improve the initiative of basic and applied research behaviors and the way they interact with each other and promote the effective integration and configuration of prosocial motivations. To this end, this study explores the applicability and configurations of various prosocial motivations in the makeup of innovation behaviors by constructing the theoretical model shown in Figure 1.

## Study design

### Data collection

Some private firms take the initiative to conduct basic research, but as public goods, basic science and the knowledge produced from it satisfy both the conditions of non-exclusivity and non-rivalry, which result in a high-risk investment for private firms. Thus, firms cannot focus on conducting basic research simply based on strong financial power (Hiromi and Koichi, 2020). Universities and public research institutes become the main body of basic research and important external providers of basic research outcomes for private firms (Chesbrough, 2003, 2006). They can conduct research independent of market mechanisms, which mostly depends on public funding. So, for employees participating in activities of basic research and basic-to-applied transformation, questionnaires were distributed to employees in universities and research institutes who were taking part in the basic research. Researchers, conducting applied science in universities, are not always able to obtain financial support for their lack of understanding on market demands. Therefore, firms and research institutes become the main body of applied research and provide important knowledge of applied research outcomes for universities. For employees participating in applied research and applied-to-basic transformation, questionnaires were distributed to employees in enterprises and research institutes who were taking part in the applied research.

Data were gathered from randomly selected employees by adopting qualitative semistructural interviews and questionnaires in two phases. The first stage of the interview with managers laid the foundation for the design of questionnaires, whereas the second stage of the interview with employees is to confirm whether the feedback of the questionnaire can be replicated in the small-scale questionnaire survey. The first stage is a small-scale pre-survey. A total of 471 questionnaires were distributed, and 210 university samples and 261 enterprise samples were obtained. After excluding invalid samples, 195 university samples and 230 enterprise samples were retained for analysis. The questionnaire was divided into five parts. The first part is to mainly understand the basic information of the respondents, and the other four parts focus on the information about employees’ prosocial motivations, basic or applied research behavior, basic-to-applied transformation behavior, and applied-to-basic transformation behavior (discussed in the section below).

The responses were measured using a five-point Likert-type scale, with the corresponding score for responses to each question survey ranging as “strongly agree” (five points), “agree” (four points), “uncertain” (three points), “disagree” (two points), and “strongly disagree” (one point). As an ethical consideration, the respondents volunteered to participate in the study and provided written consent before answering the questionnaire. They were told that they could discontinue their participation at any time without any consequences. To ensure anonymity, personal information was kept in a master file that was separate from the dataset used for the study analysis.

We chose respondents from various levels of gender, working years, professional degree, positional title, and industry. Regarding the respondents’ gender composition, men accounted for 53.86%. The respondents were grouped by professional degree, namely college diploma and below (3.76%), bachelor’s degree (29.02%), master’s degree (36.33%), and doctorate (30.9%). Although the sample had a small proportion of members with a college diploma involved in innovation activities, it was consistent with the current innovation practice. Meanwhile, participants in the other groups were evenly distributed. Employees with a high degree had more knowledge on the promotion of innovation activities, which improved the accuracy of data collection. The respondents were grouped by working years, namely 3 years and below (18.37%), more than 3 years and below 10 years (34.03%), more than 10 years and below 20 years (35.28%), and more than 20 years (12.32%). Similarly, the proportions of respondents in different industries were random. Conclusively, the questionnaire’s data collection could be deemed reliable and comprehensive.

### Research method

Qualitative comparative analysis (QCA) is a type of research method to solve complex social problems that various reasons induced (Ragin, 2008). It focuses on exploring similar or different configurations from dependent variables. Fuzzy-set qualitative comparative analysis (fsQCA) can avoid information loss and improve data accuracy in the process of data transformation. It not only integrates the quantitative research method and qualitative research method to explore the advantages of different levels of elements but also fully considers the subtle influence of different degrees of factors on the results.

### Variable measurement

The measures of prosocial motivation refer to the studies of Gebauer et al. (2008), Batson et al. (2011), and Grant and Berry (2011). Specifically, measures of pleasure-based and pressure-based motivations refer to the scale of Gebauer et al. (2008). Measures of global, contextual, and situational motivations refer to the scale of Grant and Berry (2011). And measures of egoism-based, altruism-based, collectivism-based, and principlism-based motivations draw on the scale of Batson et al. (2011). There are a total of 28 items to test prosocial motivations. After removing the trap item, the reliability (Cronbach’s α) of every scale is all above 0.7 and the factor loadings of the same variable measured in Table 3 are all above 0.55, which indicates good reliability and validity.

**TABLE 3:** Analysis of the reliability and the validity.

Item in study 1	Factor loading	Types of prosocial motivation	Cronbach’s alpha	Item in study 2	Factor loading	Types of prosocial motivation	Cronbach’s alpha
YY1	0.859	Pleasure-based type	0.7	YY1	0.851	Pleasure-based type	0.71
YY2	0.855			YY2	0.809		
LT1	0.72	Altruism-based type	0.79	LT1	0.637	Altruism-based type	0.81
LT2	0.707			LT2	0.678		
LT3	0.733			LT3	0.8		
LT4	0.626			LT4	0.718		
JT1	0.76	Collectivism-based type	0.88	JT1	0.791	Collectivism-based type	0.88
JT2	0.687			JT2	0.634		
JT3	0.838			JT3	0.84		
JT4	0.765			JT4	0.826		
XN1	0.715	Principlism-based type	0.82	XN1	0.727	Principlism-based type	0.83
XN2	0.754			XN2	0.705		
XN3	0.632			XN3	0.674		
XN4	0.641			XN4	0.67		
YL1	0.776	Pressure-based type	0.7	YL1	0.658	Pressure-based type	0.72
YL2	0.606			YL2	0.694		
YL3	0.795			YL3	0.675		
YL4	0.704			YL4	0.783		
LJ1	0.722	Egoism-based type	0.81	LJ1	0.676	Egoism-based type	0.86
LJ2	0.843			LJ2	0.832		
LJ3	0.795			LJ3	0.8		
LJ4	0.886			LJ4	0.77		
QJ1	0.68	Contextual type	0.82	QJ1	0.647	Contextual type	0.78
QJ2	0.721			QJ2	0.755		
QJ3	0.679			QJ3	0.667		
QJ4	0.624			QJ4	0.661		
QK1	0.597	Situational type	0.77	QK2	0.65	Situational type	0.79
QK2	0.622			QK4	0.683		
AR1	0.81	Basic research behavior	0.91	FR1	0.555	Applied research behavior	0.93
AR2	0.854			FR2	0.56		
AR3	0.798			FR3	0.539		
AR4	0.782			FR4	0.656		
AR5	0.654			FR5	0.766		
AR6	0.614			FR6	0.809		
AR7	0.614			FR7	0.773		
AR8	0.765			FR8	0.776		
TS1	0.874	Basic-to-applied transformation behavior	0.95	ST1	0.851	Applied-to-basic transformation behavior	0.95
TS2	0.899			ST2	0.894		
TS3	0.895			ST3	0.838		
TS4	0.889			ST4	0.821		
TS5	0.879			ST5	0.801		
TS6	0.834						
TS7	0.639						

Measures of employees’ innovation behaviors are based on the scale of Kleysen and Street (2001) and Nagane and Sumikura (2020). Specifically, the measurement items of basic and applied research behaviors refer to the scale of Kleysen and Street (2001), with a total of eight items. The reliability (Cronbach’s α) of every scale is all above 0.9 and the factor loadings of the same variable measured in Table 3 are all above 0.55. It indicates that the questionnaire results have good reliability and validity. The items of basic-to-applied transformation behavior refer to the scale of Nagane and Sumikura (2020), with a total of seven items. Combined with the similarity between applied-to-basic and basic-to-applied transformation situations, the measurement items of applied-to-basic transformation behavior are revised, with a total of five items. The reliability (Cronbach’s α) of every scale is all above 0.9 and the factor loadings of the same variable measured in the table are all above 0.55. In addition, *p*-value in the Bartlett test is less than 0.05. It also shows that the questionnaire has good reliability and validity.

## Empirical analysis

### Necessity test

Before the configuration analysis, the high level and non-high level of each condition require a necessity test (see Table 4). The condition is determined to be necessary for the result when its consistency level is greater than 0.9. The test finds that principlism-based motivation is necessary for both highly/non-highly basic research behavior and highly/non-highly basic-to-applied transformation behavior. The situational type is essential for the behaviors of highly applied research and highly applied-to-basic transformation. And the severe absence of egoism-based type is a necessary condition for non-highly basic-to-applied transformation behavior. This study retains these necessary conditions in further analysis.

**TABLE 4:** Necessity test of the previous conditions.

Condition variable	Outcome variable							
	Highly basic research behavior	Non-highly basic research behavior	Highly applied research behavior	Non-highly applied research behavior	Highly basic-to-applied transformation behavior	Non-highly basic-to-applied transformation behavior	Highly applied-to-basic transformation behavior	Non-highly applied-to-basic transformation behavior
Pleasure-based motivation	0.503	0.631	0.576	0.650	0.467	0.695	0.576	0.650
∼Pleasure-based motivation	0.789	0.844	0.762	0.789	0.771	0.850	0.762	0.789
Altruism-based motivation	0.829	0.864	0.819	0.800	0.795	0.874	0.819	0.800
∼Altruism-based motivation	0.524	0.709	0.574	0.711	0.485	0.767	0.574	0.711
Collectivism-based motivation	0.841	0.851	0.883	0.824	0.815	0.864	0.883	0.824
∼Collectivism-based motivation	0.491	0.688	0.499	0.672	0.459	0.762	0.499	0.672
Principlism-based motivation	0.918	0.902	0.848	0.818	0.90	0.927	0.848	0.818
∼Principlism-based motivation	0.390	0.599	0.549	0.700	0.369	0.672	0.549	0.699
Pressure-based motivation	0.809	0.847	0.815	0.853	0.772	0.876	0.815	0.853
∼Pressure-based motivation	0.535	0.711	0.580	0.660	0.505	0.758	0.580	0.660
Egoism-based motivation	0.555	0.677	0.620	0.720	0.530	0.722	0.620	0.721
∼Egoism-based motivation	0.771	0.852	0.767	0.782	0.741	0.900	0.767	0.782
Contextual motivation	0.875	0.818	0.847	0.771	0.820	0.826	0.847	0.771
∼Contextual motivation	0.479	0.757	0.534	0.724	0.450	0.792	0.534	0.724
Situational motivation	0.882	0.814	0.924	0.861	0.845	0.848	0.924	0.861
∼Situational motivation	0.432	0.697	0.440	0.611	0.420	0.758	0.440	0.611

### Configuration analysis

#### Configuration analysis of highly homogeneous innovation behaviors

As can be seen from Table 5, two configurations are leading to highly basic research behavior, which are combinations of “altruism-based × collectivism-based × principlism-based × pressure-based × contextual × situational” (configuration 1), and “∼pleasure-based × altruism-based × collectivism-based × principlism-based × ∼egoism-based × contextual × situational motivation” (configuration 2). The consistency and the coverage rate of configuration 1 are 0.95 and 0.61, respectively, which are higher than those of configuration 2. And both configurations 1 and 2 can explain that more than half of the innovators have this combination of prosocial motivations to realize highly basic research behavior. The common point of the two configurations is that the principlism-based type of motive plays a core role. It happens because the basic research behavior is generally decoupled from the market, and the research activities conducted are relatively independent without attaching to the interests and the needs of others. To obtain basic research achievements in a simple and exploratory environment without interests chasing, a stable personality tendency like research faith to the prosocial motivation on life and intrinsic values is very important. This finding is also shared by Liu et al. (2022) that the members of scientific research have a belief in making innovations that will have a significant impact on their attitudes toward research challenges and the knowledge interactions with other members. Another common point is that altruism-based, collectivism-based, contextual, and situational motives all assist in reaching highly basic research behavior with the core condition. The basic research behavior is a type of complex and long-term innovation behavior, so leading it to the high innovation behavior needs divergent prosocial motivations inside, which can meet employees’ spiritual pursuit in different ways for a long time. It not only needs teamwork but also allows the employees to respond selectively to the same situation and allows an employee to respond according to different situations. Including the motives above, the egoism-based type is not essential but can be optional in configuration 1, for the reason that the sense of self-achievement can be shown differently for different employees in the basic research department. This conclusion clarifies the previous conclusion that egoism-based motivation has no definite effect on basic research behavior (Tian et al., 2021), and further confirms that self-achievement motivation can be the best predictor of an individual’s high innovation behaviors in addition to professional quality and cognitive ability, but the positive effect of it on knowledge sharing weakens when employees excessively pursue success and consider the benefits of work (Higgins, 1998; Elliot et al., 2018). Configuration 1 explains more than configuration 2 because basic researchers with pressure-based motive tend to be more innovative than the researchers without pleasure-based and egoism-based motives with the premise of the same other motives working. Specifically, neither the motive to please others nor to achieve oneself excessively contributes to highly basic research behavior. But if pressure-based motivation exists, both motives can probably exist in the configuration of highly basic research behavior, for employees sometimes work for eliminating external pressure.

**TABLE 5:** Configuration solutions to highly basic research behavior and highly applied research behavior.

Types of prosocial motivation	Configuration solutions to highly basic research behavior		Configuration solutions to highly applied research behavior			
	Configuration 1	Configuration 2	Configuration 3	Configuration 4	Configuration 5	Configuration 6
Pleasure-based type		⊗		•		•
Altruism-based type	•	•	•	•	•	
Collectivism-based type	•	•	•	•	•	•
Principlism-based type	⚫	⚫	•	•	•	•
Pressure-based type	•				•	•
Egoism-based type		⊗	⊗			⊗
Contextual type	•	•	•	•	•	•
Situational type	•	•	⚫	⚫	⚫	⚫
Consistency	0.950	0.944	0.988	0.977	0.982	0.988
Original coverage	0.610	0.528	0.509	0.389	0.522	0.323
Unique coverage	0.135	0.053	0.034	0.008	0.031	0.013

The small black circle “•” indicates the presence of a secondary condition. The big black circle “⚫” indicates the presence of a core condition. And the thin circle with “⊗” indicates the absence of a secondary condition.

Four configurations lead to highly applied research behavior (see Table 5). Configurations 3–5 are relatively similar, which are the combinations of “altruism-based × collectivism-based × principlism-based × ∼egoism-based × contextual × situational,” “pleasure-based × altruism-based × collectivism-based × principlism-based × contextual × situational,” and “altruism-based × collectivism-based × principlism-based × pressure-based × contextual × situational motivation.” The coverage rate of configuration 5 is 0.522, which is the highest among the four pathways. And both configurations 3 and 5 can explain more than half of the innovators possess this combination of prosocial motivations to realize highly applied research behavior. In common, the four configurations share the situational motivation that plays a core role in highly applied research behavior. It happens because the behavior of applied research possesses the market-oriented characteristics of innovation, which is subject to the changeable market. To achieve highly applied research behavior, it requires employees to respond selectively to prosocial motivations based on the direction of market development. It is worth noting that applied researchers need to stand up and deal with unexpected situations in the research process. The result has been explored from the study by Tian et al. (2021) that applied researchers often need to invest more time, energy, and resources in their work to absorb and master domain and creative skills and form more flexible cognitive structures and in-depth strategies to deal with challenging problems. Another common point among the four configurations is that collectivism-based, principlism-based, contextual, and situational motivations all assist in reaching highly applied research behavior in the configuration. This point is the same as highly basic research behavior for applied research is also a complex activity in a long run. Configuration 5 is more convincing to explain the behavior than configurations 3 and 4, because the pressure-based motivation in configuration 5 may play a more significant role, and there exists the absence of egoism-based type in configuration 3 and the presence of pleasure-based type in configuration 4, when all other motivations work. The same as highly basic research behavior, external pressure is a good drive for applied innovators to conduct the research, for their work is challenging and should be adapted to the changing times.

Highly basic and highly applied research behaviors share in common collectivism-based, principlism-based, contextual, and situational motivations which play a supporting role in configurations. But the core conditions between the two are different. The former gets the prosocial motivation for initiative as the core condition, and the latter gets the passive motivation as the core. The most important reason this happens is that the achievements obtained from applied research meet the conditions of exclusivity and effectiveness, which is different from highly basic research behavior and may lead to knowledge hiding when conducting innovation activities. Meanwhile, applied research needs innovators to consider all aspects of the applying process and to emphasize technical cooperation to maximize benefits, which is suitable for the market behavior. However, basic research behavior is out of market behavior, as a type of innovation behavior protected and supported by the government and social organizations. So, basic research members need more intrinsically active motivations than applied research members. In addition, the number of configuration solutions in highly applied research behavior is more than that in highly basic research behavior. It gets more pathways to lead to highly applied research behavior, which is in line with the characteristics of market diversification and research types.

#### Configuration analysis of highly heterogeneous innovation behaviors

There are two configurations leading to highly basic-to-applied transformation behavior (see Table 6). Configurations 7 and 8 are the combinations of “altruism-based × collectivism-based × principlism-based × pressure-based × contextual × situational,” and “∼pleasure-based × altruism-based × collectivism-based × principlism-based × ∼egoism-based × contextual × situational motivation.” The coverage rate of configuration 7 is 0.544, which is higher than that of configuration 8. Compared with highly basic research behavior, only configuration 7 can explain that more than half of the innovators have this combination of prosocial motivations to realize this type of highly innovation behavior. It takes place because basic-to-applied transformation activity puts forward more requirements for the innovators. The first and the most important reason it happens is that basic and applied research behaviors are heterogeneous innovation behaviors. The transformation process has to overcome knowledge heterogeneity, which not only proposes some requirements for the ability and preferences of employees in the department but also can be affected by the emergent environment and emotional conditions. The second reason is that the concern of innovation behavior changes from meeting spiritual needs to cooperating with team members, and the concern of innovation achievements needs to change from being out of the market to meeting the market application. So, it is harder for innovators to achieve highly basic-to-applied transformation behavior when compared with highly basic research behavior. This finding has been verified by Sun Y. et al. (2020) that knowledge diversity and heterogeneity may harm knowledge coupling, and further confirmed by Liu et al. (2022) that transformation behavior has to invest more time, energy, and resources to achieve high innovation behavior. Besides, it is the same as highly basic research behavior that altruism-based, collectivism-based, contextual, and situational motivations all assist in reaching highly basic-to-applied transformation behavior. Although basic-to-applied transformation research is an applied research activity for a technological breakthrough, it is still based on the innovation knowledge and prosocial motivation possessed by the innovators themselves, which cannot be separated from the psychological characteristics of the ontology and innovative characteristics of the activity.

**TABLE 6:** Configuration solutions for high transformation behaviors between basic research and applied research.

Types of prosocial motivation	Configuration solutions to highly basic-to-applied transformation behavior		Configuration solutions to highly applied-to-basic transformation behavior			
	Configuration 7	Configuration 8	Configuration 9	Configuration 10	Configuration 11	Configuration 12
Pleasure-based type		⊗		•		•
Altruism-based type	•	•	•	•	•	
Collectivism-based type	•	•	•	•	•	•
Principlism-based type	⚫	⚫	•	•	•	•
Pressure-based type	•				•	•
Egoism-based type		⊗	⊗			⊗
Contextual type	•	•	•	•	•	•
Situational type	•	•	⚫	⚫	⚫	⚫
Consistency	0.953	0.956	0.907	0.915	0.900	0.943
Original coverage	0.544	0.476	0.570	0.445	0.585	0.377
Unique coverage	0.120	0.051	0.035	0.008	0.030	0.014

The small black circle “•” indicates the presence of a secondary condition. The big black circle “⚫” indicates the presence of a core condition. And the thin circle with “⊗” indicates the absence of a secondary condition.

Highly applied-to-basic transformation behavior also has four configurations (see Table 6). The coverage rate of configuration 11 is 0.585, which is the highest among the four pathways. Both configurations 9 and 11 can explain that more than half of the innovators have these kinds of personality traits and states to realize this type of high innovation behavior. They are almost the same as that of highly applied research behavior. Both of them have situational motivation which plays a core role. Common ground is difficult to develop in applied-to-basic transformation activities because crowd members lack a common organizational or situational context, show minimal commitment to pursuing a common goal, and enter or leave conversations at any point (Faraj et al., 2011; Viscusi and Tucci, 2018). So, applied-to-basic transformation behavior needs more situational motivation to achieve high innovation behavior. Among four configurations, collectivism-based, principlism-based, contextual, and situational motivations all assist in reaching highly innovation behavior. The reason it causes is that applied-to-basic transformation researchers still have the same prosocial motivation to continue the research based on the original discipline, and exert their unique motivation advantages.

Both highly basic-to-applied and highly applied-to-basic transformation behaviors share in common collectivism-based, principlism-based, contextual, and situational motivations that play a supporting role in configurations. But the core conditions between the two are different. Both of them get one prosocial motivation for initiative and passivity as the core condition, respectively. Employees participating in applied-to-basic research activities should have a certain understanding of the organization, interpretation, and perception in a particular situation, and then decide what behavior they should have (Bandura, 1999). However, employees in basic-to-applied research activities take the accountability for knowledge acquisition and regard academic achievement and honor as an important spirit pursuit, which can lead to highly basic-to-applied transformation behavior without attaching great importance to the situations (Liu et al., 2022). So, highly applied-to-basic research behavior needs more passive motivations than highly basic-to-applied research behavior.

The fsQCA method has the characteristic of asymmetry, that is, the preconditioned configuration in which a certain result appears or does not appear is not opposite. To fully explore the prosocial motivation of employees’ innovation behavior, further analysis of the condition configuration leading to a non-high level of innovation behaviors is required (see Tables 7, 8).

**TABLE 7:** Configuration solutions to non-highly basic research behavior and non-highly applied research behavior.

Types of prosocial motivation	Configuration solutions to non-highly basic research behavior		Configuration solutions to non-highly applied research behavior	
	Configuration 1	Configuration 2	Configuration 3	Configuration 4
Pleasure-based type	•	⊗	•	•
Altruism-based type	•	•	⊗	•
Collectivism-based type	⊗	•	•	•
Principlism-based type	⚫	⚫	•	•
Pressure-based type	•	•	⚫	⊗
Egoism-based type	⊗	⊗	⊗	⚫
Contextual type	•	•	•	•
Situational type	•	⊗	•	•
Consistency	0.814	0.838	0.845	0.854
Original coverage	0.554	0.497	0.520	0.505
Unique coverage	0.121	0.064	0.056	0.041

The small black circle “•” indicates the presence of a secondary condition. The big black circle “⚫” indicates the presence of a core condition. The thin circle with “⊗” indicates the absence of a secondary condition, and the bold circle with “⊗” indicates the absence of a core condition.

**TABLE 8:** Configuration solutions to non-high transformation behaviors between basic research and applied research.

Types of prosocial motivation	Configuration solutions to non-highly basic-to-applied transformation behavior	Configuration solutions to non-highly applied-to-basic transformation behavior			
	Configuration 5	Configuration 6	Configuration 7	Configuration 8	Configuration 9
Pleasure-based type	⊗	⚫		⚫	
Altruism-based type	•	•	•		•
Collectivism-based type	•	•	•	•	•
Principlism-based type	⚫	•	•	•	•
Pressure-based type			⊗	•	•
Egoism-based type	⊗		⊗	⊗	⚫
Contextual type	•	•	•	•	•
Situational type	•	•	•	•	•
Consistency	0.528	0.762	0.778	0.850	0.796
Original coverage	0.691	0.481	0.485	0.442	0.512
Unique coverage	0.047	0.003	0.028	0.008	0.029

The small black circle “•” indicates the presence of a secondary condition. The big black circle “⚫” indicates the presence of a core condition. The thin circle with “⊗” indicates the absence of a secondary condition, and the bold circle with “⊗” indicates the absence of a core condition.

#### Configuration analysis of non-highly homogeneous innovation behaviors

As can be seen from Table 7, there are two configurations that induce non-highly basic research behavior. Configurations 1 and 2 are “pleasure-based × altruism-based × ∼collectivism-based × principlism-based × pressure-based × ∼egoism-based × contextual × situational,” and “∼pleasure-based × altruism-based × collectivism-based × principlism-based × pressure-based × ∼egoism-based × contextual × ∼situational motivation” respectively. The consistency of configuration 2 is 0.838, which is higher than that of configuration 1. But the coverage rate of configuration 1 is 0.554, which is higher than that of configuration 2. It reflects that configuration 1 can explain that more than half of the innovators have this combination of prosocial motivations, which result in non-highly basic research behavior. The severe absence of collectivism-based and egoism-based motivations plays a core role in configuration 1, and the severe absence of egoism-based and situational motivations plays a core role in configuration 2. Because the basic research is the exploration of an unknown field and its research period is very long, employees with a high lack of collectivism-based and egoism-based motivations, or their high lack of both egoism-based motivation and different feedback in different situations, will go against their spiritual pursuit of self-achievements and result in non-highly basic research behavior. Thus, this finding verifies the conclusion of Elliot et al. (2018) that the severe lack of self-achievement motivation with no group thinking or situational approach results in a non-highly basic research behavior. Meanwhile, basic research activities have long-term and continuous requirements for research work, which do not seek profit, and have high requirements for knowledge dissemination and work tacit understanding, so the employees participating in it also need a sense of self-achievement to satisfy their spiritual pursuits. If the employees in the basic research department only blindly carry out altruistic activities but ignore their need, it is not conducive to a highly basic research behavior.

There are also two configurations inducing non-highly applied research behavior (see Table 7), which are configurations 3 and 4. The coverage rate of the former is 0.520, which is higher than that of the latter. And the consistency of the latter is 0.854, which slightly higher than that of the former. Both configurations can explain more than half of the innovators who have this combination of prosocial motivations conduct non-highly applied research behavior. The two configurations are quite different. The former has a severe absence of the altruism-based motivation and the presence of the pressure-based type as the core conditions, while the latter possesses a severe absence of the pressure-based motivation and the presence of the egoism-based type as the key conditions. The latter happens because the applied research behavior exists in varying degrees, and its market trend and technical cooperation lead innovators to utilitarian thought. Thus, it promotes the performance of the egoism-based type. But if the innovators focus too much on the sense of self-achievement and lose the guidance of the stress, it will lead the applied research behavior to a non-high level of innovation. The reason the former happens is that the applied research is an innovative activity fundamentally. Employees in the applied research department need a free and relaxed external environment to achieve high innovation behavior. When the environment exerts too much pressure on the innovators and they are highly lacking altruistic motivation without the egoism-based type as the secondary condition, the innovators will lose the motivation to innovate further. This finding has been proposed by Škerlavaj et al. (2018) that employees who perceive greater pressure to help others will hide knowledge when they are low in altruistic motives.

#### Configuration analysis of non-highly heterogeneous innovation behaviors

As can be seen from Table 8, there is only one configuration that induces non-highly basic-to-applied transformation behavior. The coverage rate and the consistency of configuration are 0.691 and 0.528, respectively. It can explain that 69.1% of the innovators have this combination of prosocial motivations to lead to non-high innovation behavior. In addition, the severe absence of the egoism-based type plays a core role. It happens because the lack of a sense of self-achievement will make the employees in the basic research department lose the incentive to incubate the achievement further. They may have an insufficient impetus to transform basic research achievements to applied research achievements. So to make sure the state of the egoism-based motivation inside the innovators is very important. It should not be essential but can be optional, which has been shown in the highly basic research behavior and also verified by Elliot et al. (2018).

Furthermore, as shown in Table 8, there are four configurations inducing non-highly applied-to-basic transformation behavior. First, configurations 7 and 9 are quite different, which are “altruism-based × collectivism-based × principlism-based ×∼pressure-based ×∼egoism-based × contextual × situational,” and “altruism-based × collectivism-based × principlism-based × pressure-based × egoism-based × contextual × situational motive,” respectively. The coverage rate of configuration 9 is 0.512, which is the highest among the four. Its consistency is 0.796. It means that configuration 9 accounts for more than half of the innovators to have the combination of prosocial motivations to result in this type of non-high innovation behavior. Specifically, egoism-based motivation plays a key role in configuration 9, while a high lack of pressure-based motive plays a core role in configuration 7. As an excessive focus on self-achievement and promotion prospects can lead employees into profit circles, it goes against highly applied-to-basic transformation behavior. However, if the innovators in the applied-to-basic transformation department lack a sense of self-achievement and external pressure, it will also make the innovation behavior worse. Configurations 6 and 8 are similar, namely the combination of “pleasure-based × altruism-based × collectivism-based × principlism-based × contextual × situational,” and “pleasure-based × collectivism-based × principlism-based × pressure-based ×∼egoism-based × contextual × situational motive.” They share the pleasure-based motive as a core condition, while the latter lacks the egoism-based type as a secondary condition when the altruism-based type is optional and the pressure-based type exists with the same other motive as the former. Due to the needed focus on the pleasantness and the healthy development of body and mind of others, there is a lack of efficiency and decisiveness in innovation. Such case results in non-highly applied-to-basic transformation behavior.

#### Comparison of innovation behaviors of initiative and passivity

According to the motivated information processing theory, the desires of individuals can shape the way they react to information (De Dreu, 2006; Nijstad and De Dreu, 2012). Generally speaking, the closer the innovators’ desire is to the intrinsic motivation, the lower the degree of information processing of the innovators. In other words, the closer the innovators’ desire is to the extrinsic motivation, the higher the information processing degree of the innovators, and the lower the efficiency of the innovator in making prosocial decisions.

**FIGURE 2:**
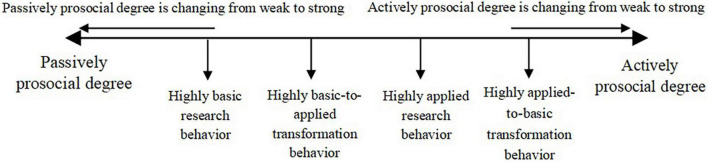
Active and passive prosocial levels of different types of high innovation behaviors.

Overall, in the comparison of configuration solutions of highly homogeneous innovation behaviors, there are four configurations in highly applied research behavior and only two configurations in highly basic research behavior. However, there is a core condition of actively prosocial motivation of principlism-based type in highly basic research behavior, but a core condition of passively prosocial motivation of situational type in highly applied research behavior. So, highly basic research behavior needs more actively prosocial motivations than highly applied research behavior. Furthermore, since the configuration solutions of highly heterogeneous innovation behaviors are similar to that of highly homogeneous innovation behaviors, highly basic-to-applied transformation behavior also needs more active prosocial motivations than highly applied-to-basic transformation behavior. In addition, both configurations in highly basic research behavior can explain that more than half of the innovators have this combination of prosocial motivations to realize highly basic research behavior, but only one configuration in highly applied research behavior can explain that. It means that highly basic research behavior needs more active prosocial motivations than highly basic-to-applied transformation behavior, and highly basic-to-applied transformation behavior needs more active prosocial motivations than highly applied research behavior. Then, because the explanation of the configurations of prosocial motivation in highly applied-to-basic transformation behavior is less powerful than that in highly applied research behavior, the former needs less active prosocial motivations than the latter. Consequently, the actively prosocial degree of different types of high innovation behaviors from strong to weak are highly basic research behavior, highly basic-to-applied transformation behavior, highly applied research behavior, and highly applied-to-basic transformation behavior (see Figure 2). It has been explored by Škerlavaj et al. (2018) that prosocial motivation and intrinsic motivation have a significant influence on knowledge sharing. They found that in organizational knowledge management, the more consistent the dominant prosocial motivation of knowledge contributors is with their intrinsic motivation, the higher the knowledge sharing level of knowledge contributors will be, and consequently, a low level of knowledge hiding.

In the comparison of configuration solutions of non-highly homogeneous and heterogeneous innovation behaviors, the former has more solutions of the absence of passive motivations in the configurations than the latter. So non-highly homogeneous behaviors for innovation depend on more passively prosocial motivations than non-highly heterogeneous behaviors. Meanwhile, within the homogeneous configurations, non-highly basic research behavior has two configurations which lack one or two passive prosocial motivations and only one initiative prosocial motivation in each configuration, while non-highly applied research behavior also has two configurations which lack one or zero initiative prosocial motivation and one passive prosocial motivation in each configuration. Hence, the passively prosocial degree of different types of non-high innovation behaviors from strong to weak are non-highly basic research behavior, non-highly applied research behavior, non-highly applied-to-basic transformation behavior, and non-highly basic-to-applied transformation behavior.

## Discussion and implications

### Discussion

Prosocial motivation plays an important role in employees’ innovation behaviors by improving the interaction between basic research and applied research. Based on the research type decomposition, this study investigated the influence of prosocial motivations on employees’ innovation behaviors. On one hand, prosocial motivations include not only motivations for initiative but also motivations for passivity. On the other hand, according to the production of innovation achievements, innovation behavior can be specifically divided into basic research behavior, applied research behavior, basic-to-applied transformation behavior, and applied-to-basic transformation behavior. Among them, basic research behavior and applied research behavior are homogeneous innovation behaviors, whereas basic-to-applied and applied-to-basic transformation behaviors are heterogeneous innovation behaviors. The results can be shown in four significant findings.

First, highly basic and highly applied research behaviors share in common collectivism-based, principlism-based, contextual, and situational motivations which play a supporting role. But the core conditions between the two kinds of research are the prosocial motivation for initiative as core condition and the motivation for passivity as core condition, respectively.

Second, both highly basic-to-applied and highly applied-to-basic transformation behaviors share the same core conditions and secondary conditions with highly basic and highly applied research behaviors, respectively, because high transformation behaviors still require innovators in different fields to have the same prosocial motivation and to continue the research based on the original discipline foundation.

Third, the behaviors of non-highly basic research and non-highly basic-to-applied transformation share the absence of egoism-based motivation as the core condition in common. But the former has some absent conditions of motivations to lead to non-high innovation behavior. Then the common point between the behaviors of non-highly basic-to-applied and non-highly applied-to-basic transformation is the absent condition of pressure-based motivation as the key condition. But the former’s severe lack of altruism-based motivation leads to non-high innovation behavior in transformation research.

Fourth, we also analyze active and passive prosocial degree of all types of high/non-high innovation behaviors. The actively prosocial degree of different types of high innovation behaviors from strong to weak are highly basic research behavior, highly basic-to-applied transformation behavior, highly applied research behavior, and highly applied-to-basic transformation behavior. The passively prosocial degree of different types of non-high behaviors from strong to weak are non-highly basic research behavior, non-highly applied research behavior, non-highly applied-to-basic transformation behavior, and non-highly basic-to-applied transformation behavior.

### Theoretical implications

Our research takes a step toward resolving the controversy about the link between prosocial motivations and innovation behaviors. Although the effect of prosocial motivation on innovation behavior has been widely explored (Gebauer et al., 2008; Pian et al., 2019), little research has addressed active and passive prosocial degree of different types of innovation behaviors. We proposed and found that this relationship is contingent on the configurations of prosocial motivations. For innovators who have strongly active prosocial motivations, configuration with some intrinsic motivations like principlism-based motivation is good for them to conduct basic research or basic-to-applied research. Meanwhile, they may have weakly passive prosocial motivations. If innovators have weakly active motivations, for example, the configuration has passive motivation like pressure-based motivation, it may result in non-highly basic research behavior. Therefore, the configuration of prosocial motivations helps researchers explain the reasons why prosocial motivation cannot lead to innovation behavior and extends the explanation of prosocial motivation.

In addition, our research presents a new relational view of innovation by considering the production of innovation achievements. Although several researchers have studied the effect of prosocial motivation on basic and applied research, they are conducted separately (Hoever et al., 2012; Kim and Choi, 2018), without considering how prosocial motivation affects different behaviors when they interact with each other. So, we discuss the impact of prosocial motivation not only on different types of innovation behaviors but also on the interaction between them. Configurations of prosocial motivation help researchers explore the way to overcome the difference between the psychological characteristics of innovators within the research departments and that of innovators between the research departments. Our study finds out that employees with highly basic research behavior or highly applied research behavior have different configurations of prosocial motivations. The difference in the configuration of employees’ prosocial motivations also can be shown in different types of highly heterogeneous innovation behaviors. Hence, our findings enrich the studies of innovation behaviors from the perspective of prosocial motivations.

### Practical implications

In addition to being of theoretical interest, our findings shed light on the practice of prosocial motivations and innovation behaviors for organizations and their employees. The conclusions of the research will help managers in different research activities understand the internal mechanism of stimulating employees’ innovation behaviors from the perspective of the combination of prosocial motivations. We suggest that simply considering a single type of prosocial motivation may not be enough, particularly when the problems being solved are ill-structured, such as strategic formulations. Our contribution to research on innovation behavior is to identify the effects of multi-dimensional prosocial motivations as a specific mechanism of knowledge coupling.

To achieve high innovation behaviors from the perspective of multi-dimensional prosocial motivations, innovation activities of all kinds can share the same practical implications in some aspects. It is necessary to create an innovative atmosphere, such as periodically organizing brainstorming and regular meetings and setting a good example to encourage employees to make research with excellence, which can promote the learning and sharing of knowledge and experience among employees. What is more, employees should be provided with as much organizational support as possible such as encouraging them to participate in training and lectures on research methods and attending national and international professional conferences to enhance professional knowledge and skills (Lee et al., 2020; Shie et al., 2020). The above methods can enhance their active prosocial motivation. Moreover, employees in all research departments need to recognize their identity and value based on the organization to engage in creative work more effectively. In addition, managers also need to allow them to participate in more research projects and enrich their work experience, which can encourage them to set higher achievement goals. Most importantly, leaders can also continuously strengthen their positive evaluation of group identity to further motivate their achievement motivation and independent innovation.

The conclusion also tells us about the breakthrough of differentiated management among different innovation activities. The leaders of the scientific research activities should pay attention to psychological incentives for innovators to meet their pursuit of spiritual rewards, whereas the leaders of the applied research activities should focus on situational management to diversify incentive methods to meet the prosocial needs of innovators, for the members in the applied research activities need more situational prosocial motivation. So, to motivate innovators to engage in more innovation activities, innovation behaviors should be encouraged in a way that incorporates various kinds of incentives and tolerances for differences and heterogeneity, as the information system not only acts as an enabler but also shapes the innovation outcomes (Majchrzak and Malhotra, 2013). In this regard, using different prosocial motivations appropriately and strategically will be the key to encouraging innovators’ prosocial interaction in the innovation process (Boudreau et al., 2011; Boudreau and Lakhani, 2015; Lakhani, 2016).

### Limitations and future research

Although this study has produced interesting findings and contributed to both theory and practice, it has several limitations. First, we analyzed texts from innovation behavior without discerning the industry sector or other environmental and market contexts. As such, we do not know how idea integration is affected by contextual conditions. We also did not have data on innovators’ industry experience and expertise, and therefore, do not know how that would affect the relationships we found. Second, the results of the study are based on data in the Chinese context, which may limit the generalization to other countries. Future research should use data from diverse countries to verify the validity of our results. Finally, our focus is solely on prosocial motivation as the source of innovation behavior. Individuals can become involved in innovation behavior to gain personal benefits, personal intrinsic rewards, or due to other proself motives. In this study, due to our interest in prosocial motivation, we did not develop other directions. We strongly encourage future researchers to delineate a separate model of proself motivation leading to various outcomes through innovation behavior and explore its boundary conditions.

## Conclusion

Considering that one dimension with difference changes the whole process of employees’ innovation behavior, the influence of multiple prosocial characteristics on innovation behaviors is complex and the causes of high and non-high innovation behaviors cannot be reversed. Accordingly, we regarded prosocial motivations as a whole, took prosocial motivation as the antecedent of innovation behavior, and constructed a model by integrating prosocial theory with innovation behavior theory to discover multiple and complex causality relationships between condition configurations consisting of various prosocial types and innovation behaviors to ensure conclusion universality. We discovered that the same level of innovation behavior depends on the configuration that consists of various prosocial types rather than a certain motivation. A certain configuration of motivations may produce different levels of certain innovation behavior. Multiple and complex causality relationships exist between condition configurations consisting of various prosocial types and innovation behaviors, which enlighten us on how to strengthen the positive effects and avoid negative effects of prosocial types on innovation behaviors to provide practical inspiration for the training of innovation talents.

## Data availability statement

The original contributions presented in this study are included in the article/supplementary material, further inquiries can be directed to the corresponding author.

## Author contributions

YL: conceptualization, methodology, and writing – review and editing. BZ: writing – original draft preparation, review and editing. LZ: investigation, data validation, and supervision. WL: software and data curation. All authors contributed to the article and approved the submitted version.
